# Spectral Performance of Multilayer Amorphous Selenium and Selenium–Tellurium Photodetectors

**DOI:** 10.1021/acsaom.4c00475

**Published:** 2025-02-25

**Authors:** Hamid Mirzanezhad, Kaitlin Hellier, Max Teicheira, Shiva Abbaszadeh

**Affiliations:** Department of Electrical and Computer Engineering, University of California—Santa Cruz, Santa Cruz, California 95064, United States

**Keywords:** photodiodes, amorphous selenium, selenium−tellurium, chalcogenide alloys, multilayer

## Abstract

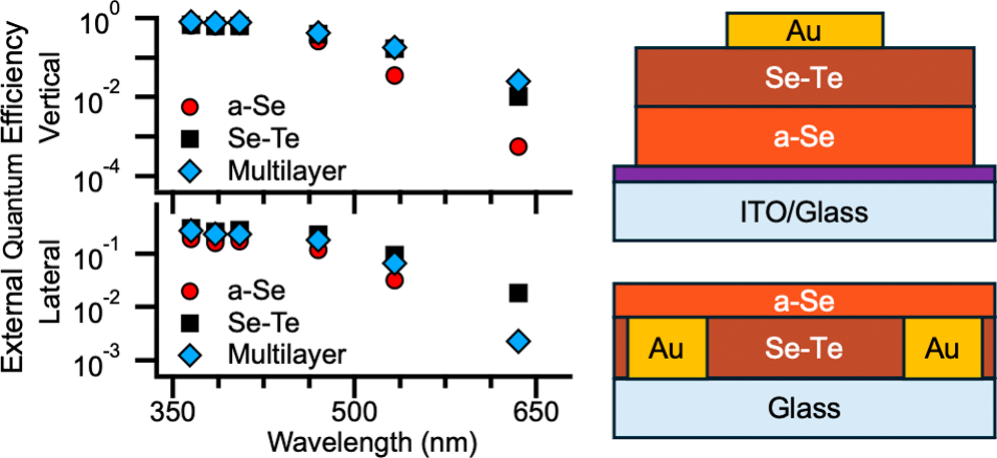

Photodiodes are an essential semiconductor device used in medical imaging, high-energy physics, and UV–visible sensors. Recent progress has renewed interest in exploring alloys of traditional materials for detector fabrication. Alloying amorphous selenium (a-Se) with other materials can potentially improve device performance in responsivity and quantum conversion efficiency (QCE) and address some limitations of stabilized a-Se. To increase the sensitivity and transport properties, we explore multilayer devices with vertical and lateral architectures. We use different combinations of stabilized a-Se and selenium–tellurium (Se–Te) alloys and compare implementing each as the light-absorbing layer, aiming to determine whether tailoring the alloys based on the wavelength absorption depth could improve the detector’s performance. For vertical devices, a thin (90 nm) a-Se layer paired with a thick (15 μm) Se–Te layer proved to be the most effective device, improving both the response at long wavelengths and overall QCE, with a 13–15% improvement over single-layer a-Se devices in the UV and 2.5% improvement at red wavelengths. In the lateral devices, the combination of a-Se and Se–Te layers outperformed a single layer of stabilized a-Se; however, a solid layer of Se–Te gave the highest QCE with a peak efficiency of 30% at 355 nm and 15 V/μm. These findings demonstrate how multilayer structures can affect device performance, better guiding device architecture based on the end application, desired wavelength sensitivity, and efficiency.

## Introduction

Amorphous selenium (a-Se) has long been studied for ultraviolet (UV), visible, and X-ray detection. Initial studies into a-Se in the 20th century focused on its xerographic properties and later shifted to its use as a material for direct and indirect conversion X-ray detectors.^1−3^

Indirect conversion detectors employ scintillator materials to convert X-ray photons to visible light. This light is then detected by photodiodes or other light-sensitive elements and converted to a digital signal. a-Se shows significant potential as a vacuum ultraviolet (VUV) to visible detector due to its high absorption coefficient across those spectra.^4^ It is especially well suited for VUV detection, making it ideal for high-energy physics applications such as liquid noble gas detectors.^5−9^

Amorphous selenium exhibits several favorable attributes, including low dark current, high conversion efficiency, and its ability to achieve impact ionization at fields above 70 V/μm, despite its low mobility and high resistivity.^10−12^ The fields for impact ionization are significantly lower than amorphous silicon, which requires fields greater than 110 V/μm, making it advantageous for applications utilizing avalanche multiplication.^13^ Additionally, a-Se can be uniformly deposited on large surface areas, with the capability of achieving thicknesses up to 1000 μm by thermal evaporation.^14^

Alloying has commonly been studied and implemented in Se to enhance its properties.^2,15,16^ For applications in indirect conversion X-ray and UV–vis detection, there is a need to detect a broader range of wavelengths than stabilized a-Se can sense. When a-Se is alloyed with Te, the band gap is reduced, increasing its sensitivity at longer wavelengths.^17^ Previous studies, including work from our group, have revealed that doping a-Se with Te improves absorption in the green-to-red wavelengths, especially at high fields, although it decreases mobility, and increases dark current and ghosting.^18−24^

In addition to the material composition, the performance of photodetectors is influenced by the device layout. Many designs have been investigated, including simple architectures such as vertical and lateral layouts and those more complicated such as Frisch grids and field-shaping multi-well avalanche detector (SWAD) structures.^25−28^

In this study, we utilize simple vertical and lateral structures to evaluate the effects of alloying on solid and multilayered material compositions. Vertical devices arrange the material layers in a stacked configuration, with the electric field oriented perpendicular to the surface, resulting in a uniform field across the thickness of the a-Se layer.^29−32^ However, short-wavelength light can be heavily attenuated by the substrate, electrical contact, and blocking layers before it can reach the photoconductor, limiting the application of vertical structures in capturing UV light.^23,33^ In contrast, lateral devices are composed of interdigitated electrodes with the semiconductor layer deposited above and between electrodes, where the field runs parallel to the substrate. In this architecture, a-Se can directly absorb incident photons at its surface and results in enhanced absorption of short-wavelength light, broadening the spectrum these devices can effectively detect.^34^

Previous work on high-gain avalanche rushing amorphous photoconductor (HARP) detectors has demonstrated the benefits of incorporating a thin Se–Te absorber layer within a multilayer device composed primarily of a-Se.^35,36^ The HARP’s increase in sensitivity over conventional charge-coupled devices (CCDs) highlighted the promising role a Se–Te layer could play in enhancing device performance.^20,37^ These works demonstrate the benefit of multilayer devices; however, they only focus on structures with thin Se–Te and a thick a-Se layer and in a vertical structure.

In this work, we evaluate the properties of both vertical and lateral device structures, examining multilayered depositions with thin a-Se or Se–Te absorbing layers to enhance the spectral response of the photodetectors. We evaluate the absorption capabilities and charge transport capabilities of devices with different architectures. Our findings and underlying mechanisms are discussed in detail, providing insights into potential improvements and future applications.

## Methods

### Device Fabrication

**Figure 1:**
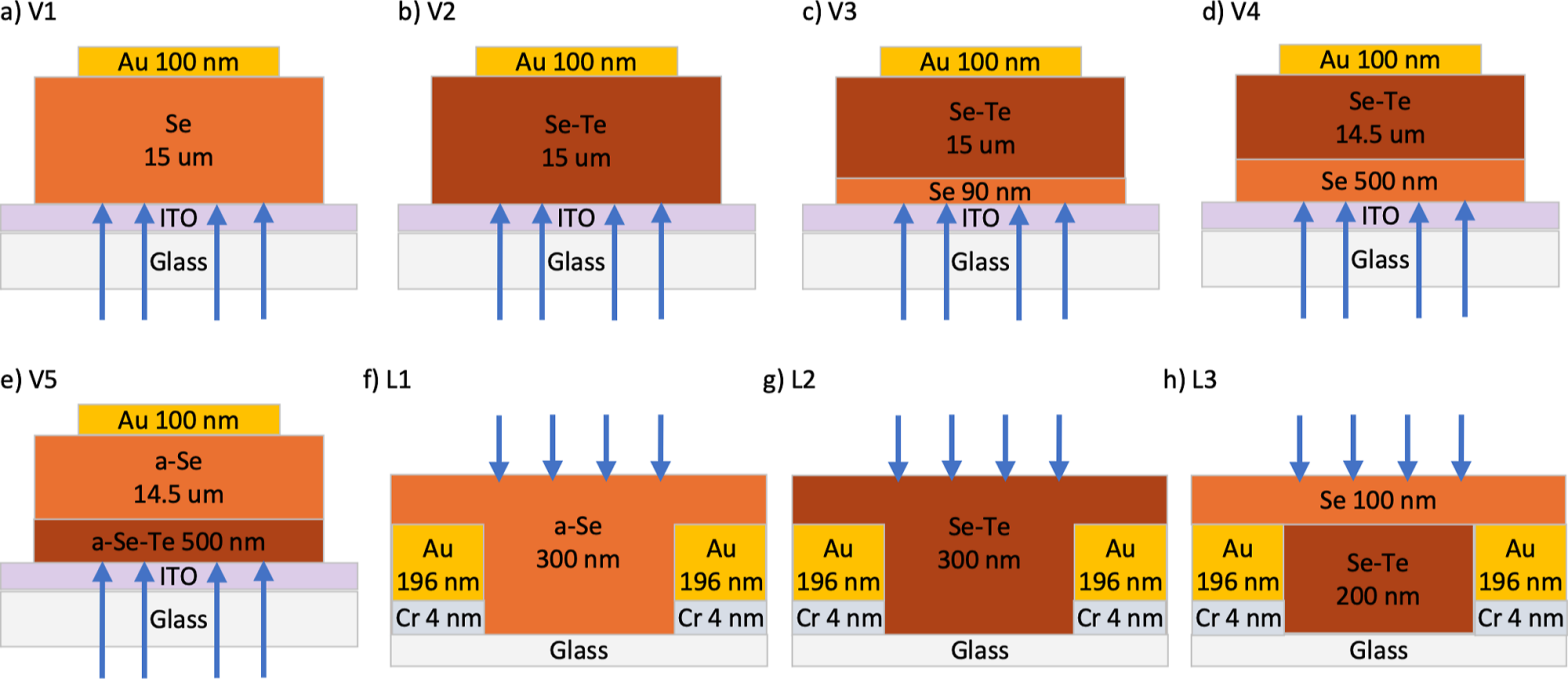
Layouts of the vertical and lateral device architectures used in this work for (a) V1, (b) V2, (c) V3, (d) V4, (e) V5, (f) L1, (g) L2, and (h) L3. The direction of incident light is indicated by arrows, with light passing through the glass/ITO for vertical devices and directly onto the semiconductor for lateral devices.

Before material deposition, all substrates were cleaned by ultrasonication in acetone and isopropyl alcohol for 10 min each, then rinsed with deionized water, and dried with nitrogen. Photoconductive layers were deposited by the thermal evaporation of stabilized a-Se (0.2% As, 10 ppm of Cl) and Se alloyed with 10 wt % Te (99.999%) on glass/ITO substrates. Electrical contacts (Cr and Au) were deposited by electron beam evaporation. Five vertical (V1–V5) and three lateral samples (L1–L3) were fabricated, with structures seen in Figure 1.

Electron-dispersive spectroscopy (EDS) was performed on films to determine elemental concentrations using an Apreo scanning electron microscope. X-ray diffraction (XRD) was performed on Se and Se–Te samples with a Rigaku SmartLab diffractometer.

### Vertical Devices

Stabilized a-Se (V1) and a-Se_0.9_Te_0.1_ (V2) single-layer samples were fabricated with a target thickness of 15 μm on an ITO/glass substrate. The first two-layer sample (V3) comprised a 90 nm a-Se base layer with a 15 μm a-Se_0.9_Te_0.1_ top layer. Sample V4 was constructed with a 500 nm stabilized Se base and a 14.5 μm a-Se_0.9_Te_0.1_ top layer. Sample V5 mimics sample V4—swapping the top and bottom photoconductor materials—with a 500 nm a-Se_0.9_Te_0.1_ base topped with a 14.5 μm stabilized a-Se layer. All samples were completed with 100 nm Au top contacts, forming 14 devices of 3, 4, and 5 mm diameters. All sample structures for V1–V5 are demonstrated in Figure 1a–e.

### Lateral Devices

Lateral devices were fabricated with single-layer and multilayer structures. The interdigitated electrodes were fabricated by photolithography and had an electrode width, *w*, and separation, *s*, of 15 μm with a total device size of 1 mm. Each slide hosts nine devices with contact pads extending to the edge of the substrate. Electrical contacts consisted of a 4 nm Cr adhesion layer topped with 196 nm Au deposited on a glass substrate. After patterning the photoresist, samples underwent 30 s of cleaning with radiative ion etching prior to electrode deposition. Post-evaporation, substrates with electrodes were additionally cleaned by ultrasonication. Photoconductive materials were deposited on top of the electrodes. Sample L1 consisted of 300 nm of stabilized a-Se (Figure 1f), L2 consisted of 300 nm a-Se_0.9_Te_0.1_ (Figure 1g), and L3 was composed of 200 nm a-Se_0.9_Te_0.1_ and 100 nm a-Se (Figure 1h).

### Device Characterization

Sample thickness, *d*, was measured by stylus and optical profilometry for the vertical and lateral devices, respectively. Lateral device thicknesses were also measured by cross-sectional SEM on a Quanta 3D FEG; final thicknesses were averaged from the results. Absorbance, *A*, for lateral devices was taken from films deposited on glass during the sample fabrication by using a Jasco V670 spectrophotometer. The absorption coefficient was extracted as α = *A*/*d*. This was combined with data taken from photothermal deflection spectroscopy, previously reported by Hellier et al., to provide the absorption coefficient from 350 to 900 nm.^23^ The penetration depth for each material was calculated as δ = 1/α.

**Figure 2:**
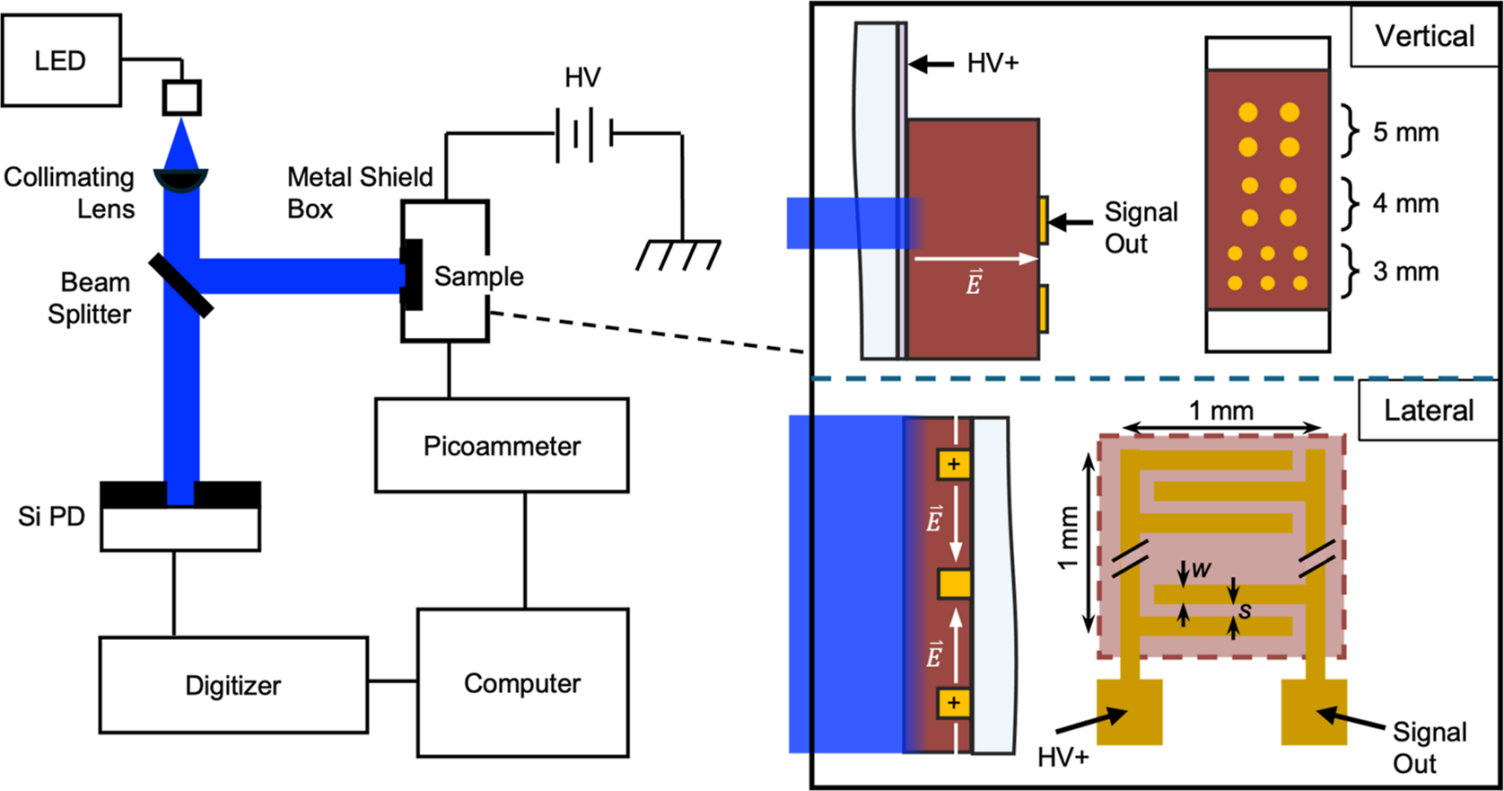
Schematic of the current measurement setup, illustrating the configuration of the LED light source, beam splitter, Si photodetector for LED irradiance monitoring, and the arrangement for directing light onto the sample. Key components and their interconnections are depicted to show the flow and measurement process. Sample connections and interaction with the incident LED light are depicted on the right, highlighting sample layouts.

Dark and photocurrent measurements were used to characterize the devices. A schematic of the optical setup can be seen in Figure 2. Low-power stabilized LEDs (Ocean Insight) with wavelengths from 365 to 635 nm were used for photocurrent measurements. The LED light was collimated and directed through a beam splitter. Half of this collimated light was used to monitor power using a Si photodetector and digitizer (Thorlabs), while the other half was directed onto the sample, which is held in a metal shield box to reduce noise. The signal was read out with a Keithley 6487 picoammeter and Kickstart 2 software. An inset demonstrates the passage of light to the vertical and lateral samples and how electrical connections are made to each sample. QCE was calculated using the equation
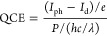
1where *I*_ph_ is the photocurrent, *I*_d_ is the dark current, *P* is the incident optical power on the photoconductor, *h* is Planck’s constant, *c* is the speed of light, *e* is the elementary charge, and λ is the wavelength of the incident light. For vertical devices, the incident power factors in absorption are a function of wavelength from the substrate. A more detailed explanation of these experiments and how information is extracted from the data can be found in Hellier et al.’s study.^38^

### Simulation

The electric field in all device architectures was simulated by using COMSOL 6.1 in two dimensions. Vertical devices were simulated with a 1 μm thick layer of glass, 75 nm ITO, photoconductor layers according to the architecture specified in Figure 1, and a 100 nm gold top contact. The glass, ITO, and photoconductor layers were 1 mm wide; the top contact was 0.5 mm wide. ITO was held at 225 V and the gold at 0 V. As the devices are symmetrical through their cross section, only one edge of the device was analyzed. For lateral devices, the widths of gold electrodes were 15 μm, placed 15 μm apart. Bias was applied at 0 and 225 V, alternating with each electrode. The photoconductor layer thickness was 300 nm, with structures designated by the specifications from above. The relative permittivities, ϵ_r_, used were 6.3 for Se and 7.7 for Se–Te. Neither the glass nor air was included in the simulated results for the lateral devices.

## Results and Discussion

### Results

Devices were fabricated with single- and multilayer structures of stabilized a-Se and 10% atomic weight Te-alloyed a-Se. Previous studies on Te content have demonstrated that higher levels of Te lead to increased hole and electron trapping, resulting in reduced carrier mobilities and lifetimes, and increased ghosting and lag at lower fields.^19,23,39^ We opted to utilize 10% weight Te, as these negative impacts are minimal, while benefits from the reduced bandgap are still notable. The photoconductor thickness of the vertical devices was chosen according to that most commonly studied in the literature in recent years; while the devices can be fabricated to thicknesses specific to photon penetration depth, we targeted 15 μm for ease of comparison with other works.^36,38,40^ Lateral devices were fabricated with a target photoconductor thickness of 300 nm with potential applications for thin film and flexible detectors in mind, along with ease of fabrication in the photolithography process.

**Figure 3:**
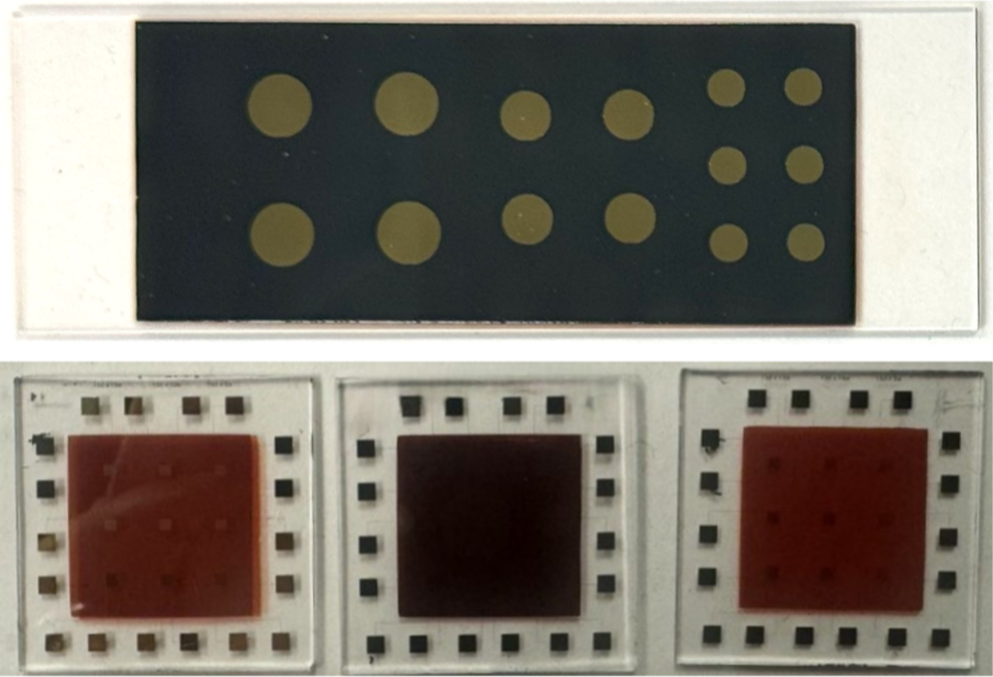
Images of fabricated samples: (top) a single vertical sample, V2, comprising 14 devices with diameters of 5 mm, 4 mm, and 3 mm, and (bottom) three lateral samples (from left to right: L1, L2, and L3), consisting of 9 devices each.

**Figure 4:**
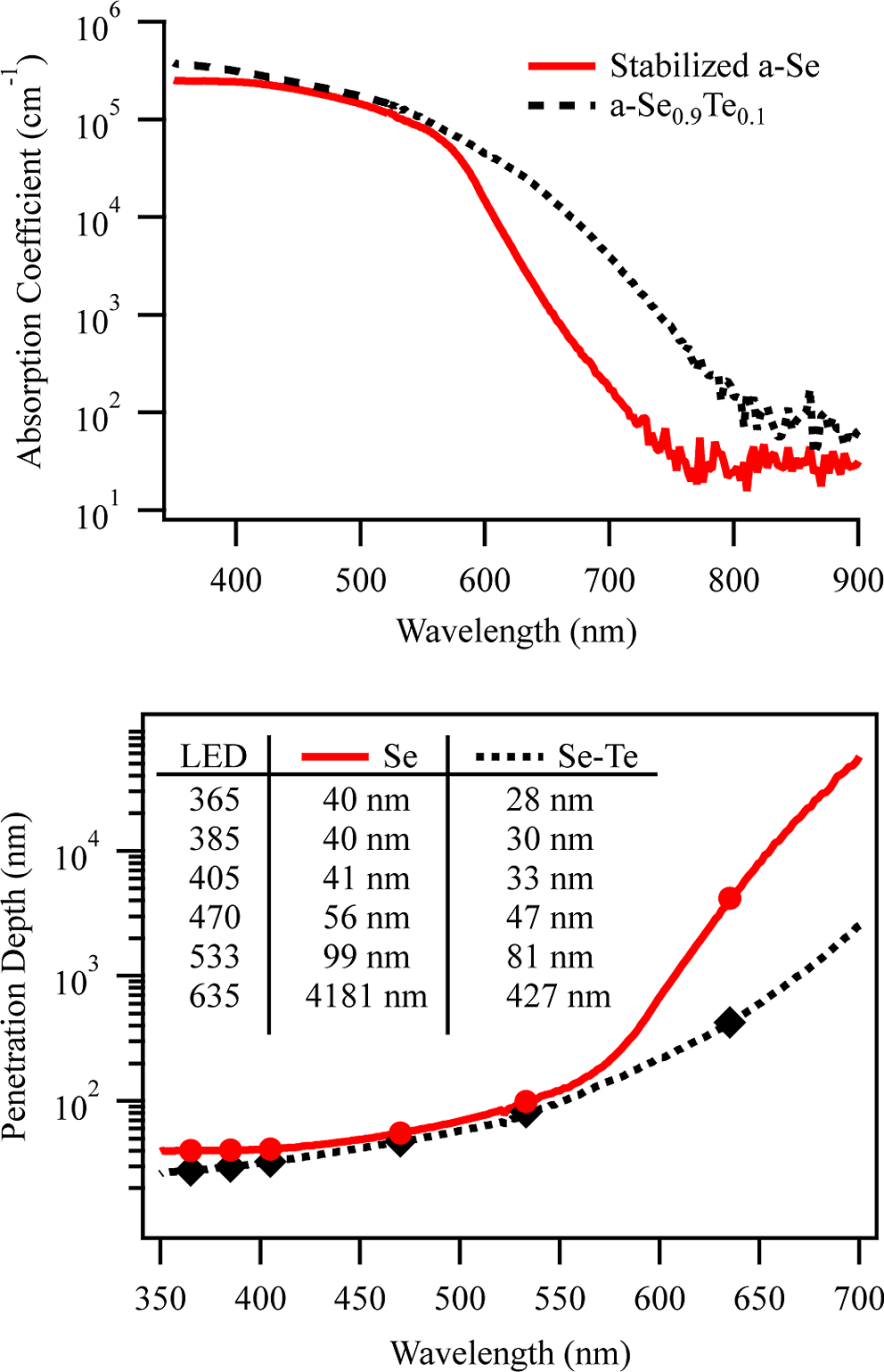
(a) Absorption coefficients for a-Se and Se–Te materials across wavelengths. (b) Penetration depth of a-Se and a-Se_0.9_Te_0.1_, with the inset graph specifying the calculated values for LEDs used in this work.

Images of a single vertical and three lateral fabricated samples are shown in Figure 3. The vertical device shows the exposed ITO substrate at the edges with gold contacts on top of the dark photoconductive layer. The lateral device has a square photoconductive layer on top of the 9 devices, with contact pads exposed along the edges. To understand how different wavelengths of light will interact with the solid and multilayer devices and to select the ideal thicknesses for multilayer fabrication, absorbance measurements for a-Se and a-Se_0.9_Te_0.1_ were performed. The absorption coefficients for these materials can be found in Figure 4a.

From this, we modeled the penetration depth, which is calculated as the point at which the intensity of light in the material reaches 1/*e* the surface intensity and is defined as δ = 1/α, where α is the wavelength-dependent absorption coefficient. This provides an estimate of the thickness through which light passes before being absorbed. Figure 4b shows the penetration depths for both a-Se and a-Se_0.9_Te_0.1_. These values are in line with those extracted from Gilleo and Lanyon for a-Se and a-Se_0.75_Te_0.25_.^41,42^ a-Se_0.9_Te_0.1_ shows a much shorter penetration depth for longer wavelengths, which agrees with its reduced bandgap. We can determine that a vertical device with an a-Se_0.9_Te_0.1_ absorbing layer should have full absorption of wavelengths at 635 nm and less under 500 nm of thickness. To limit long-wavelength absorption from a-Se for improved QCE from the a-Se_0.9_Te_0.1_ layer, the a-Se thickness should be limited to no more than 100 nm, leading to the selection of 90 nm for our a-Se absorption layer for the a-Se/Se–Te device. To compare to devices used in other studies with thicker layers, we also fabricated devices with a 500 nm absorber layer.

The fabrication process, as illustrated in Figure 1, resulted in devices with final thicknesses that were measured, as shown in Table 1. These thicknesses, while closely aligned with our target values, exhibited some variation from the intended target. These deviations can be primarily attributed to several factors. In all devices, the differing *Z*-factors, which refer to the atomic number (*Z*) of the material being deposited, of Se compared to Se–Te contributed to inconsistent deposition rate monitoring, impacting the final thicknesses of the layers. For vertical devices, this had the greatest impact. In the lateral devices, the high deposition rate used to maintain amorphous behavior with optimal transport makes precise deposition of very thin layers difficult as the final thickness is reached in less than 10 s of the deposition phase.^43^

**Table 1:** Sample Naming Conventions, Layer Configurations, and Final Thicknesses for the Devices Used in This Work^a^

sample	architecture	layer 1/layer 2	thickness
V1	vertical	15 μm Se	14.45 μm
V2	vertical	15 μm Se–Te	13.35 μm
V3	vertical	90 nm Se/15 μm Se–Te	16.4 μm
V4	vertical	500 nm Se/14.5 μm Se–Te	14.1 μm
V5	vertical	500 nm Se–Te/14.5 μm Se	18.2 μm
L1	lateral	300 nm Se	340 ± 15 nm
L2	lateral	300 nm Se–Te	335 ± 15 nm
L3	lateral	200 nm Se–Te/100 nm Se	337 ± 10 nm

a All vertical device measurements have a standard error of 0.15 μm.

Concentrations for the stabilized Se and Se–Te were found to be within error of the intended values and with homogeneous elemental distribution. Images and analysis of these results can be found in Figure S1.

Cross-section SEM images of the lateral devices showed homogeneous material deposition with no voids or cracking and gave electrode width and separation within error of the intended values. The high resistivity of both Se and Se–Te gave poor resolution in the SEM images, resulting in higher measurement errors than typically expected from SEM, limiting the analysis performed. These images and additional discussion can be found in the Supporting Information.

Analysis of XRD measurements on both Se and Se–Te vertical devices, shown in Figure S3, showed amorphous behavior, which presents as a broad peak around 24° and with no sharp peaks, indicating crystallinity. Repeat measurements on samples showed consistent behavior among different depositions.

### Vertical Devices

**Figure 5:**
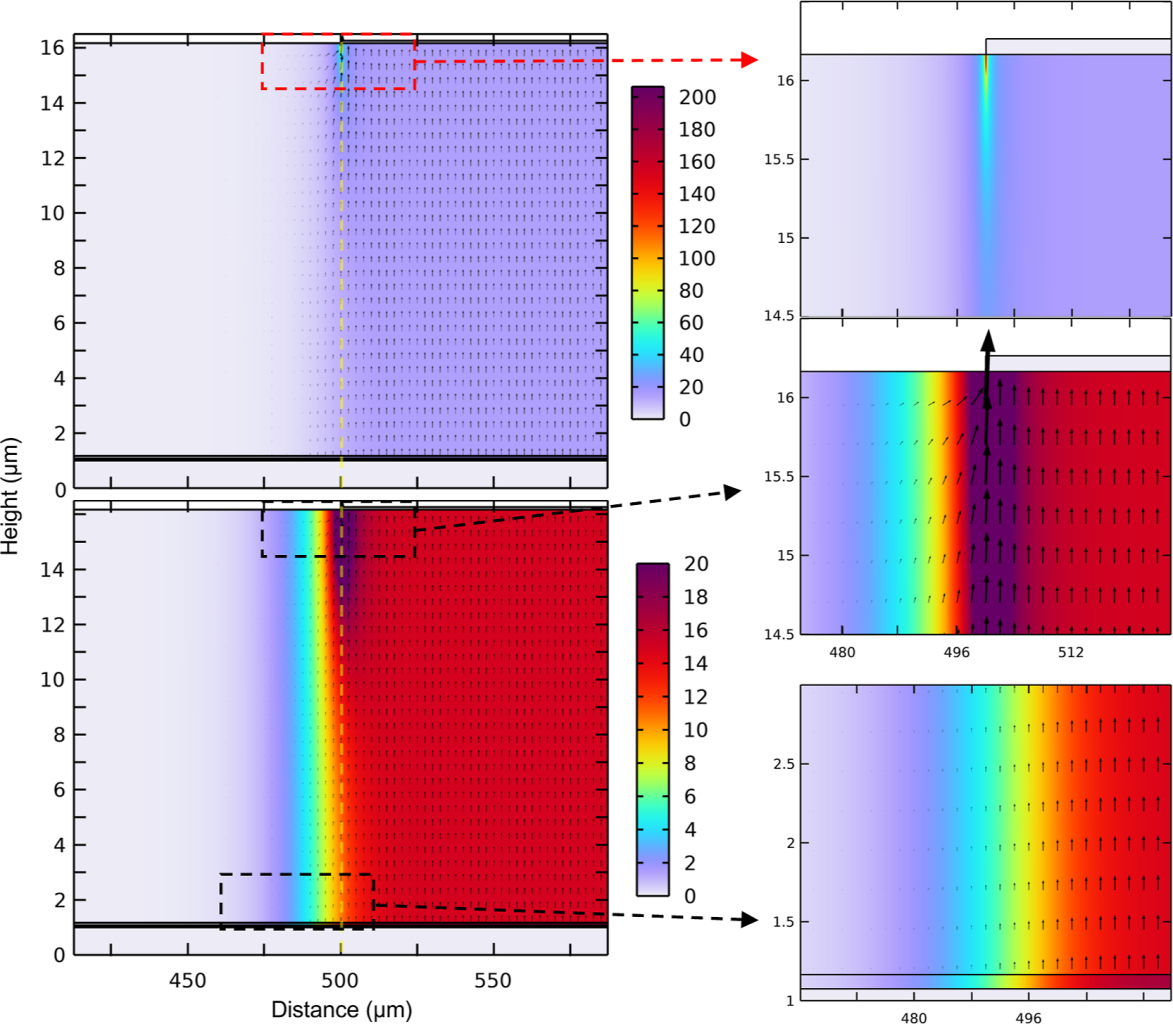
COMSOL models of device V3—90 nm a-Se with 15 μm Se–Te—focusing on the region between the edge of the top electrode and the ITO/glass substrate. Color scales indicate field strength, with arrows indicating the strength and direction of the electric field. The top left and right plots utilize a color scale from 0 to 208 V/μm, highlighting edge effects around the top electrode. Arrows have been omitted from the top right plot to prevent obscuring details of the field variation. The bottom left and middle and bottom right plots utilize a color scale from 0 to 20 V/μm to highlight the small changes in the field at the border of the active area of the device and between the a-Se and Se–Te layers, which show a difference of over 2 V/μm.

Simulations of the electric field in vertical device architectures biased at 225 V were modeled by using COMSOL. Figure 5 shows highlighted portions of the simulation for sample V3, focused around the region between the edge of the top electrode and the ITO/glass substrate. The top left and right images depict a color scale highlighting the uniformity and edge effects around the electrode, while the bottom left and middle and bottom right depict a color scale to highlight the variances at lower field levels. Arrows indicate the field strength and direction. In all architectures, the electric field remains constant between the electrodes through the bulk of the material, falling off quickly after the termination of the top electrode, within 20 μm. There is a slight edge effect at the border of the top electrode, highlighted in the top right of Figure 5, where the electric field spikes to 208 V/μm; however, this can be noted to be very minimal in size, on the order of nanometers relative to the millimeter scale of the device.

Focusing on the layers of a-Se and Se–Te, we do not see any interface effects. However, we do note that the change in relative permittivity between the materials leads to a shift in the field across the two materials, as highlighted in the bottom right of Figure 5. While our single-layer devices, V1 and V2, maintain a field of 15.0 V/μm across the entirety of the semiconducting layer, for V3 and V4, we see that the thin a-Se layer has an increased field of 17.3 V/μm and the thick Se–Te layer a slightly reduced field of 14.9 V/μm; for V5, we see a drop in our thin Se–Te layer to 13.0 V/μm and a slightly higher than expected field in the thick a-Se layer of 15.1 V/μm. Plots for samples V1, V2, V4, and V5 can be found in the Supporting Information.

Vertical devices were characterized by dark current density and quantum conversion efficiency—calculated from eq 1—as a function of field and incident wavelength. Quantum efficiency across a range of fields was investigated at the wavelength of 365 nm, and a field of 15 V/μm was used to characterize the response at wavelengths from 365 to 635 nm.

**Figure 6:**
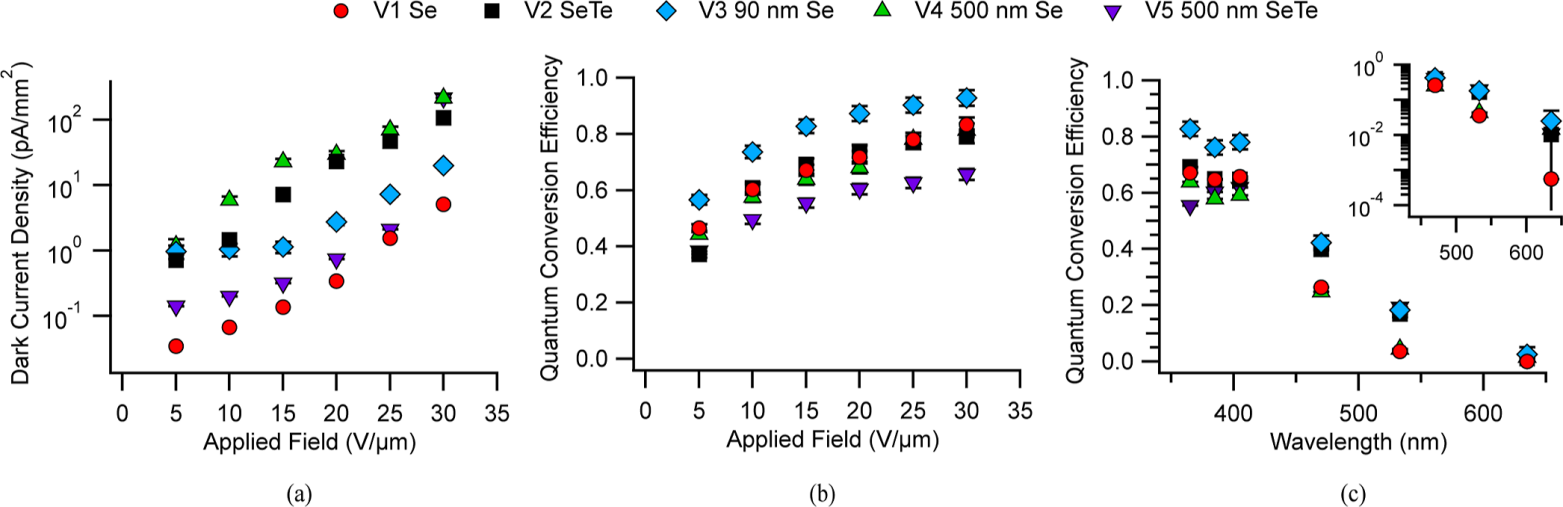
(a) Dark current density of devices V1–V5 as a function of increasing electric field. (b) Quantum conversion efficiency of the devices from 5 to 30 V/μm at a wavelength of 365 nm. (c) The QCE response of devices at 365 to 635 nm at an applied field of 15 V/μm; the semilog inset highlights the increased sensitivity of Se–Te absorbers at longer wavelengths.

As seen in Figure 6a, the dark current increases when the applied field increases (as expected), and the V2 and V4 samples are higher than the other structures. It is known that alloying Te into a-Se increases conductivity and dark current density, so this is not surprising.^44^ As expected, the V1 device has the lowest dark current, while the V3 device shows intermediate levels compared with the other devices.

In Figure 6b, the QCE results for V1 and V2 increase with increased field, in line with previous studies.^23,45^ V3 outperforms the others, possibly due to its architecture, and V4 follows the trends of V1 and V2. V5 underperforms compared with the others, indicating that a Se–Te thin film before a-Se reduces the extraction of UV light.

As shown in Figure 6c, device performance at wavelengths above the band gap of a-Se follows similar trends, with V3 performing better at 365, 385, and 405 nm. Other devices fall within error of each other, besides the lower performance of V5 at 365 nm.

Below the band gap of a-Se, we see the effects of Te alloying. The wavelength-dependent response of the V2 device shows improved performance compared with the V1 device. This improvement is attributed to the lower band gap of a-Se_0.9_Te_0.1_ in the V2 device, which enhances long wavelength performance.^42^ Samples V2, V3, and V5 have a higher response at longer wavelengths, highlighted in the semilog plot inset, as a result of Se–Te absorption. V3 has thin Se, allowing longer wavelengths to pass through and be absorbed by the a-Se_0.9_Te_0.1_ layer. V2 and V5 have Se–Te absorber layers, directly converting the long wavelengths. V1 and V4 have a-Se absorption layers, preventing longer wavelengths from passing through and resulting in a lower response of Se at longer wavelengths.

### Lateral Devices

Previous studies on lateral devices have shown that the electric field extends only between electrodes, with minimal to no field over the electrodes themselves.^25^ To demonstrate this for our devices, COMSOL models with 15 μm electrodes separated by 15 μm with a 300 nm active layer of a-Se, Se–Te, or a multilayer of 200 nm Se–Te/100 nm a-Se were developed.

**Figure 7:**
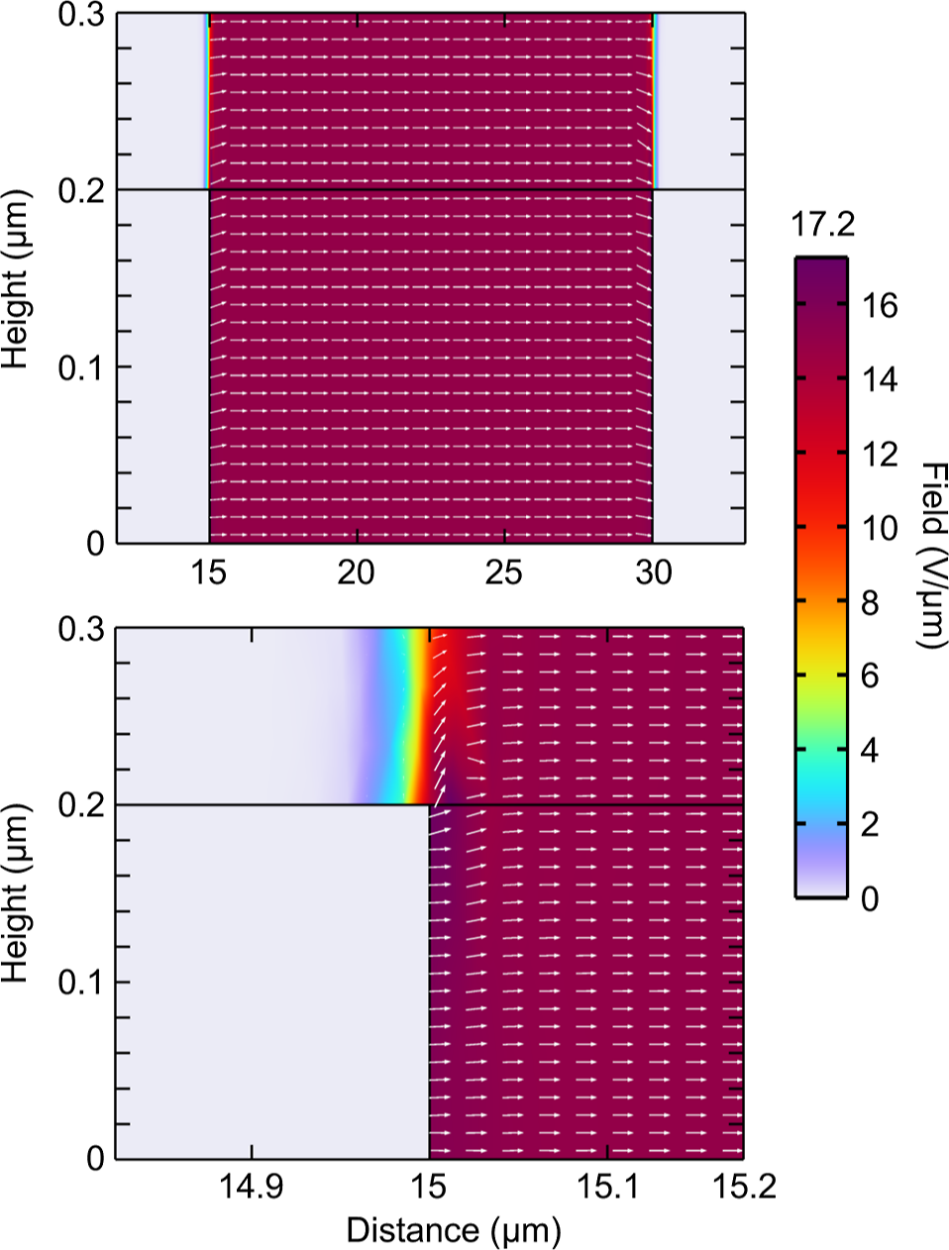
Simulation of the electric field across the lateral devices, performed with COMSOL. Top shows the full area between electrodes; bottom shows the region of ±200 nm around the edge of the positive electrode. Arrow size and thickness indicate the strength and direction of the electric field, along with a color scale for emphasis on the field strength, which demonstrates that the field over the electrode reduces to zero within 50 nm from the edge and a slight increase in the field at the corner of the electrode.

As expected, we see the field fade quickly from 15 to 0 V/μm within 50 nm from the edge of the electrode, as shown in Figure 7. The field maintains a uniform distribution throughout the a-Se layer between the electrodes up to the majority of the a-Se, with a small increase to 18 V/μm at the top corner of the electrode, less than 50 nm from its edge. This tells us that the active area of the device is limited to the area where no electrodes are present, reducing our area from 1 to ∼0.488 mm^2^. We also see a slight reduction of the field at the top edge of a-Se.

The model did not show any differences for Se–Te or for the multilayer device, as seen in Figure S5 of the Supporting Information. Additionally, no interface effects were present in the multilayer model, likely due to the similar relative permittivities of Se and Se–Te.

Incorporating insights from our simulation, we experimentally explored the behavior of the lateral devices. Dark current density and QCE versus the applied field, as well as the QCE at different wavelengths (365–635 nm at 15 V/μm), were characterized with the light directly incident on the photoconductive layer.

**Figure 8:**
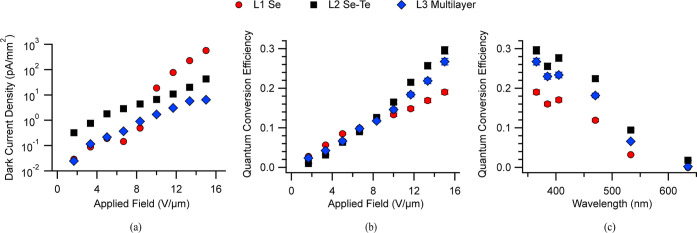
(a) Dark current of devices L1–L3 as a function of the electric field. (b) Quantum conversion efficiency of the same devices as a function of the electric field, taken using a 365 nm LED. (c) QCE of L1–L3 from 365 to 635 nm at a field of 15 V/μm.

As shown in Figure 8a, dark current density measurements exhibit trends different from those observed in vertical devices. The solid a-Se device (L1) has current densities lower than those of the other two devices at low fields but experiences a sharp increase around 10 V/μm, a pattern consistently observed across multiple tests and samples. The solid Se–Te lateral device (L2) shows higher leakage than both V3 and the initial L1 results, which matches the increased conductivity of Se–Te previously reported.^42^ The combination of Se and Se–Te layers in (L3) initially displays a current comparable to that of V1 and remains low even after L1 increases at 10 V/μm. This suggests that the increased current from the a-Se layer is potentially suppressed by the Se–Te layer, preventing the jump observed in the a-Se device.

For the lateral devices, we quantify the QCE as external quantum efficiency (EQE), which does not account for the reflection and scattering of photons at the incident surface, unlike the QCE measurements for vertical devices. The photocurrent values for L1 align with those reported in previous studies on lateral devices, assuming a linear response to light intensity and considering the specific device areas.^46^

As shown in Figure 8b, all devices follow a similar trend at fields below 10 V/μm. Starting at 10 V/μm, all devices continue an upward trend; however, L2 and L3 increase at a greater rate, with L2 outperforming L3, which outperforms L1. The combination of Se and Se–Te layers in L3 gives an intermediate result consistent with the performance of each layer.

The response of the structures at 15 V/μm, found in Figure 8c, follows a similar pattern; L2 performs best, L3 performs slightly lower, and the L1 device falls short of both. L3 maintains some of the improved performance from Se–Te, with higher long-wavelength performance compared to L1. Device L1 did not register a response from 635 nm light, which is consistent with the device’s thickness, light penetration depth, and high dark current.

### Discussion

Dark currents and QCE values at 15 V/μm are summarized for the vertical and lateral devices in Table 2. Vertical devices highly outperform lateral devices, which is consistent with what has been observed in other studies. We can see that V3 outperforms all other devices across all wavelengths by 12–15% in the UV range and is on par with other Se–Te devices at long wavelengths. Sample L2 outperforms other lateral devices; it, however, falls short of vertical devices by a significant amount in the UV and by about half at longer wavelengths. In the following sections, we will discuss the possible reasons and meanings for these results.

**Table 2:** Dark Current Density and QCE at Each Wavelength for Each Device Biased at 15 V/μm^a^

sample	*J*_d_ (pA/mm^2^)	365 nm	380 nm	405 nm	470 nm	533 nm	633 nm
V1	0.136 ± 0.135	0.672	0.646	0.658	0.264	0.036	<0.001
V2	7.141 ± 0.247	0.692	0.650	0.648	0.399	0.168	0.0103
V3	1.127 ± 0.223	0.827	0.762	0.780	0.422	0.182	0.025
V4	22.725 ± 2.512	0.639	0.579	0.591	0.248	0.044	0.015
V5	0.317 ± 0.229	0.554	0.603	0.619	0.410	0.193	0.011
L1	574.700 ± 26.041	0.190	0.160	0.171	0.119	0.032	--
L2	42.901 ± 3.914	0.296	0.256	0.277	0.224	0.094	0.018
L3	6.492 ± 0.132	0.268	0.230	0.234	0.182	0.066	0.002

a Standard error on QCE, which combines errors in alignment, intensity, and average photocurrent value, is given as 0.020.

### Vertical Devices

**Figure 9:**
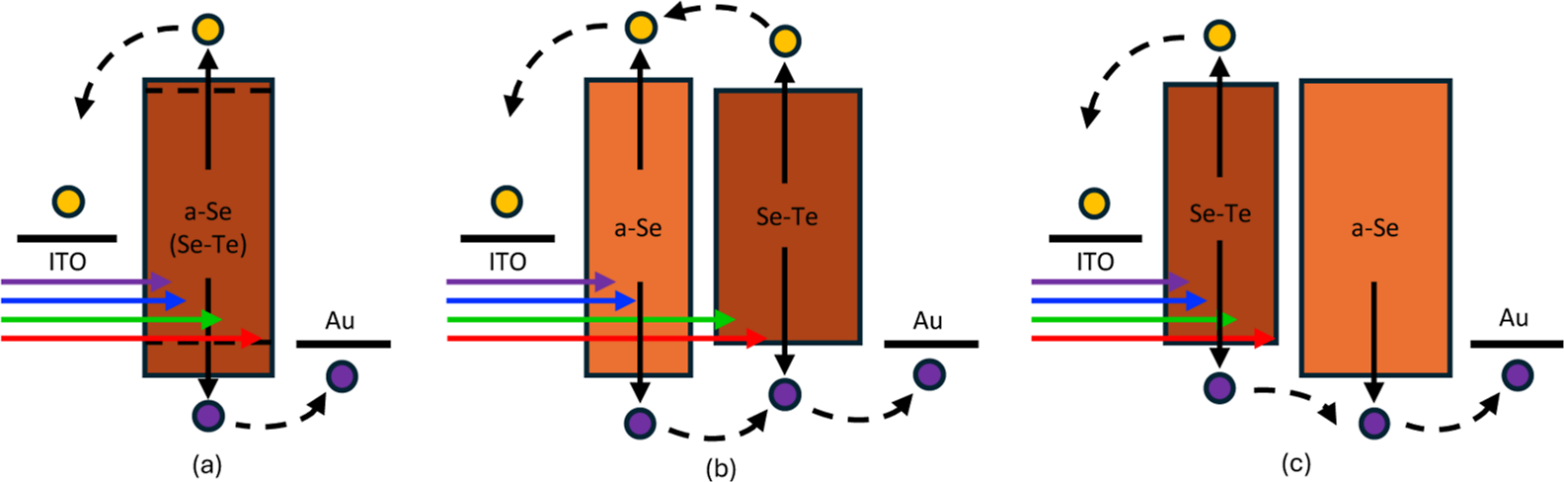
A cartoon schematic of the band energies for different layer configurations, illustrating the absorption of various wavelengths and carrier transport for (a) solid a-Se or Se–Te, (b) multilayer devices with a-Se as the first layer, and (c) multilayer devices with Se–Te as the first layer.

To understand the behavior of the vertical devices, we propose the simplified band schematic in Figure 9 to demonstrate the interactions between the a-Se and Se–Te layers. Transport in a-Se is understood to be a multiple-trapping hopping mechanism, with extended and localized states playing a large role in carrier extraction.^47^ Additionally, the charge generation and collection efficiency are known to be field-dependent, especially in the region around 15 V/μm, as demonstrated in this work and previous studies.^45^ In an a-Se absorption layer device (V3 and V4), the increased electric field across a-Se, noted from the COMSOL simulations, will increase charge generation for the photons absorbed in that layer; the wider gap of a-Se also helps to transport holes from the a-Se to the Se–Te layer. The reduced gap of Se–Te will reduce the Schottky barrier at the Au interface, further improving extraction.^48^ Conversely, employing a Se–Te absorption layer (V5) reduces the field in the charge-generating region and creates an additional energy barrier for the transport of holes, potentially reducing performance.^49^ The variance in the ITO and Au work functions, along with the Se and Se–Te interface between the materials, will lead to a built-in electric field in the device, potentially creating reduced or greater barriers between the Se–Te. In addition, the gradual shift from Se to Se–Te (or vice versa) and possible diffusion at the interface will lead to a shift in transport. Future work will delve deeper into understanding this interface along with the shift in band energies due to Te inclusion.

We see the greatest performance in device V3, possibly due to employing a thin Se layer, which minimizes the time for the loss of high-energy carriers from extended to localized states and more effectively reaching the Se–Te layer. This, combined with the increased field, reduced Schottky barrier, and limited absorption from Se at long wavelengths (allowing them to pass to the Se–Te), gives improved QCE across all wavelengths. The slight increase in dark current relative to V1 and V5 can be considered a reasonable trade-off for the increase in QCE and may be reduced in applications by the inclusion of charge-blocking layers.

V4 performs similarly to V1 and V2 at short wavelengths as the carriers have more time to relax in the Se layer, losing energy and potentially falling into localized states before reaching the Se–Te layer. Its performance drops at long wavelengths because the light is primarily absorbed by Se, where longer wavelength light has lower charge generation and is more susceptible to localized states, limiting carrier extraction into the Se–Te layer.

At 365 nm, V5 may underperform due to the reduced electric field across the Se–Te and additional barriers introduced. Holes generated in the Se–Te layer relax to states around the band edge but then require more energy to hop into a-Se states. At long wavelengths, V5 shows similar performance to V2 and V3 as the carriers are excited into extended rather than localized states and are able to reach the extended a-Se states during transport through the Se–Te, consistent with observations in previous studies.^24,37^

### Lateral Devices

As each layer connects to the metallic contact directly, the band schematic does not apply here; each material is mostly subject to its own transport properties.

Dark current values for the devices follow expected trends, except for L1, which is the solid a-Se device. In studies of other thin film lateral a-Se devices, we have observed higher-than-expected dark current values, bringing into question whether the change in the orientation of the applied field relative to the growth direction of the a-Se plays some role in transport. A recent study by Lu et al. theorizes that heat and light exposure lead to the shift from a ring to a chain structure; if correct, a chain structure perpendicular to the electric field may lead to different transport through the material.^50^ Further study on this is required, with greater emphasis on how the thickness of a-Se plays a role in how carriers may be transported across the surface of a-Se.

All lateral devices show a lower QCE across comparable electric fields and incident wavelengths than the vertical device; this is consistent with previous findings.^46^ The reasoning for the drop in QCE for lateral devices is well observed but not well understood and may again be related to a shift in transport mechanisms relative to the orientation of the electric field.

Devices with Se–Te show increased performance at longer wavelengths, as expected from the reduced band gap. The thinner layers utilized will limit full absorption at long wavelengths, reducing the observed QCE. The photocurrents found in L1, the solid a-Se device, are in line with those found by Abbaszadeh et al.^46^ Device L3, our multilayer structure, shows a combined performance of L1 and L2. Given the thin layer of Se and a thicker layer of Se–Te, it should be expected that its performance is better than a 50/50 combination of Se and Se–Te performance. Though Se has a low penetration depth for most of the wavelengths, some of the light will still pass through to the Se–Te, which will fully absorb the remaining light, except at 635 nm. The reduced Schottky barrier for Se–Te may play a role in improved performance for devices L2 and L3; again, more study of lateral transport through these materials is required to fully understand their behavior.

Ultimately, at these thicknesses, the use of a lateral multilayer device does not show a benefit to device performance. This finding emphasizes the importance of carefully considering the device architecture and material thickness in designing multilayer optical detectors. We may achieve a better response for all materials by applying higher fields, which could be assisted by employing a blocking layer or by increasing the thickness of the a-Se_0.9_Te_0.1_ layer. However, this approach may lead to an undesired drop in efficiency from the a-Se layer as it sits further from the electrodes, and carriers will be transported through the Se–Te. On the other hand, it may help in reducing dark currents if surface conduction in a-Se is the cause.

## Conclusions

In this study, we explored a-Se/Se–Te multilayer photodetectors by exploring different architectures and layer variations. The Se–Te alloy, specifically in the a-Se_0.9_Te_0.1_ combination, increases absorption at longer wavelengths and improves charge transfer. This enhancement in the wavelength response and quantum efficiency aligns with previous studies.

In vertical devices, an a-Se/Se–Te multilayer structure outperforms a-Se_0.9_Te_0.1_, demonstrating that adding 90 nm of a-Se before a Se–Te layer results in increased quantum efficiency relative to a solid Se–Te layer due to an enhancement in charge generation and transport in the layers. In lateral devices, the solid Se–Te outperforms other devices, and our findings show that multilayer devices do not have any advantage over other devices, which is counter to what we may have anticipated. This and previous studies indicate that there may be more complicated transport mechanisms occurring where the field is applied perpendicular to the growth direction of the photoconductive layer. However, more in-depth material studies must be conducted to draw any conclusions.

These findings demonstrate the potential and pitfalls of multilayer photodetector architectures in enhancing device performance. The success of vertical multilayer structures in improving sensitivity and efficiency, especially in configurations optimized for specific wavelength ranges, illustrates the importance of design for high-performance optical detectors. Understanding the strengths and limitations of different multilayer architectures provides a path for making decisions when designing detectors with specific application requirements.

## Supporting Information Available

EDS and cross-sectional SEM images of Se and Se–Te devices; XRD of Se and Se–Te; and additional COMSOL models for the vertical and lateral devices (PDF)
