# Supplementary material for: Spectral Performance of Multilayer Amorphous Selenium and Selenium–Tellurium Photodetectors
Source: ACS Appl Opt Mater. 2025 Feb 25;3(3):646–55. doi: 10.1021/acsaom.4c00475 (PMC11959609; doi:10.1021/acsaom.4c00475)
Supplement: Supplementary file 1 — ot4c00475_si_001.pdf [file ot4c00475_si_001.pdf]

Supporting Information:
Spectral performance of multi-layer amorphous selenium and selenium-tellurium photodetectors

Hamid Mirzanezhad, Kaitlin Hellier, Max Teicheira, and Shiva Abbaszadeh*

Department of Electrical and Computer Engineering, University of California - Santa Cruz, Santa Cruz, California, 95064, USA

E-mail: sabbasza@ucsc.edu

Cross-sectional scanning electron microscopy (SEM) and energy dispersive spectroscopy (EDS)

Composition of the bulk films was determined by employing scanning electron microscopy (SEM) energy dispersive spectroscopy (EDS). Films demonstrated uniform distribution of elements, as seen in the representative images in Figure S1. For vertical devices, only the top layer of material was able to be analyzed; measurements were not expected to penetrate beyond 5 um. For the lateral devices, arsenic levels in the stabilized a-Se were expected to be skewed, as some arsenic may be present in the glass substrate; the multilayer lateral device data was not measured, as it was excepted to be too skewed by the substrate.

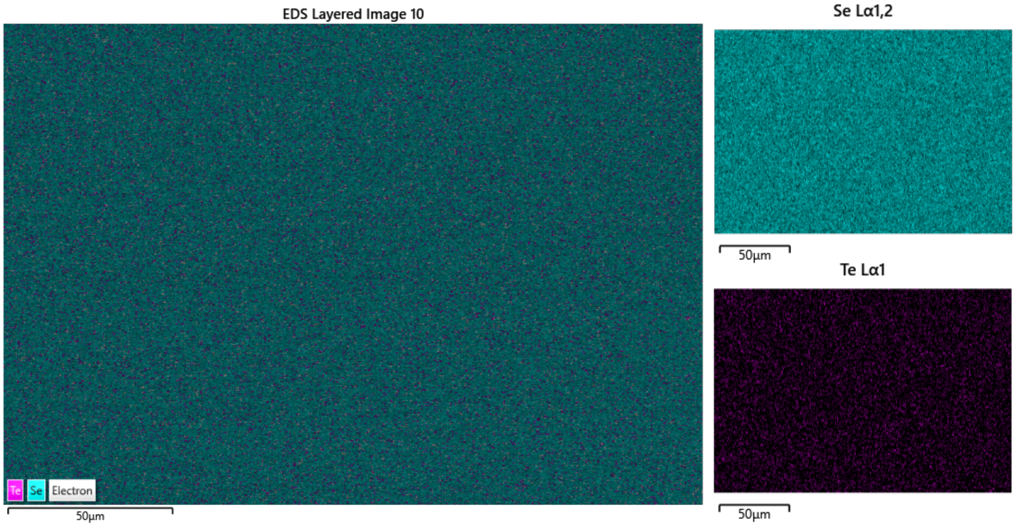

Figure S1. Energy dispersive spectroscopy images of sample V2, showing uniform distribution of Se and Te throughout the sample. The left image shows the combined spectra, with individual elements shown on the right for Se (top) and Te (bottom). Elemental analysis for this sample showed a Se concentration of 89.7wt. % and Te concentration of 10.3 wt. %.

All materials fell within ±0.8% of their intended target, which also falls within the error of the instrument employed. For Se-Te alloys, Te concentrations fell within 9.5-10.8 weight (wt.) %; For selenium stabilized by doping with As, values for As concentration ranged from 0.1-1.2 wt. %, including lateral devices. It has been noted during this and other studies by this group that, when employing now well-developed fabrication recipes for the Se-Te, a consistent and repeatable ratio of Se to Te can be achieved. The material used for stabilized selenium is pre-alloyed, giving repeatable results and performance.

Cross-sectional SEM was performed on lateral devices to ensure electrodes were well fabricated and a-Se and Se-Te morphology appeared similar to previous studies. Images of these cross sections can be seen in Figure S2.

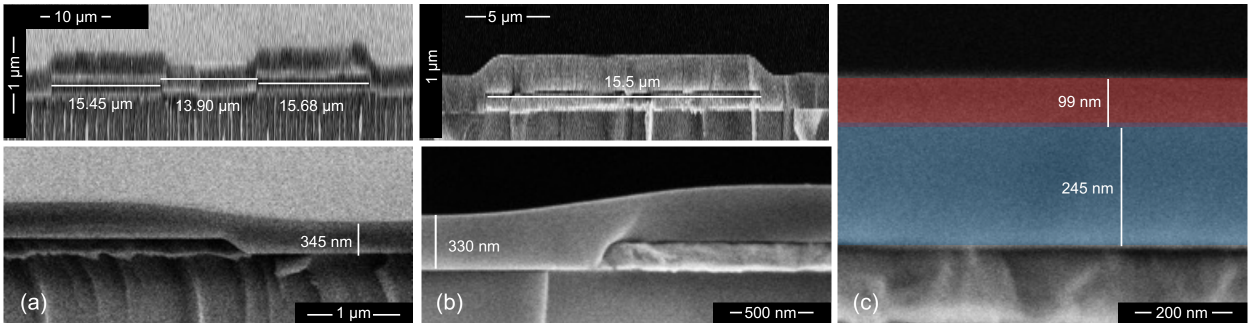

Figure S2. Cross-sectional SEM images of a) L1 (solid selenium) and b,c) L3 (multilayer) devices. a) (Top) Image of two electrodes in the L1 sample, showing electrode width and spacing, with an error of ± 0.5 um. (Bottom) Image of one electrode edge, showing the slight slope around the edge of the electrode and the thickness of the a-Se, ± 15 nm, in the active area. b) (Top) Image of one full electrode an L3 sample demonstrating the electrode width, ± 0.5 um. (Bottom) Image of the multilayer sample around one electrode edge, and thickness of the multilayer in the active area, ± 10 nm. c) Image of the multilayer over one electrode, in which each layer has been recolorized (with a slight overlap) indicating the two layers and their thicknesses.

A slight slope can be seen along the edge of the electrodes, as seen in Figure S2a and S2b, indicating some deviations in the lift off process during photo lithography. Electrode widths measure 15.5 um, further supporting additional removal of resist during the photolithography process. Thickness measurements are similar to those measured by optical profilometry, however, given the high resistance of Se and Se-Te, sharp edges can be difficult to distinguish. Additionally, the amorphous nature of these materials tends to result in slight lipping of the material over the break edge, further obscuring an accurate measurement. Figure S2c shows a colorized cross-section of the multilayer film over an electrode. After manipulation of the original image – recoloring and contrast adjustment – a region of transition between the Se and Se-Te became distinguishable. This region showed an area of change, highlighted by the overlap in the colorized image above. This is not surprising, given the similar compositions of the materials and

overlap of Se that would obscure a clear transition between materials and create a blended region. All films appear homogenous, with no signs of voids or cracking.

Amorphous behavior and stability

X-ray diffraction (XRD) was performed to ensure amorphous behavior in the samples after fabrication and prior to testing. Figure S3 shows XRD scans from a stabilized a-Se sample and a Se-Te sample. The broad spectra and lack of peaks show amorphous behavior for both materials, in line with previous reports. All samples exhibit this behavior, and is easily repeatable following fabrication recipes.

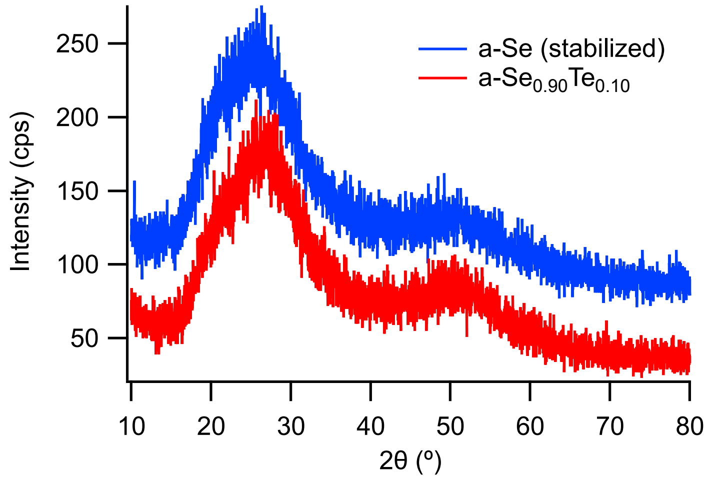

Figure S3. XRD of a stabilized a-Se sample and a Se-Te sample, demonstrating amorphous behavior through a lack of sharp peaks and a low intensity, broad peaks around 25° and 50°, seen in glassy materials. For ease of viewing, the stabilized a-Se has been shifted up by 50 cps.

COMSOL Simulation

COMSOL models were created to simulate the electric field between electrodes in vertical and lateral devices for the structures utilized in this work.

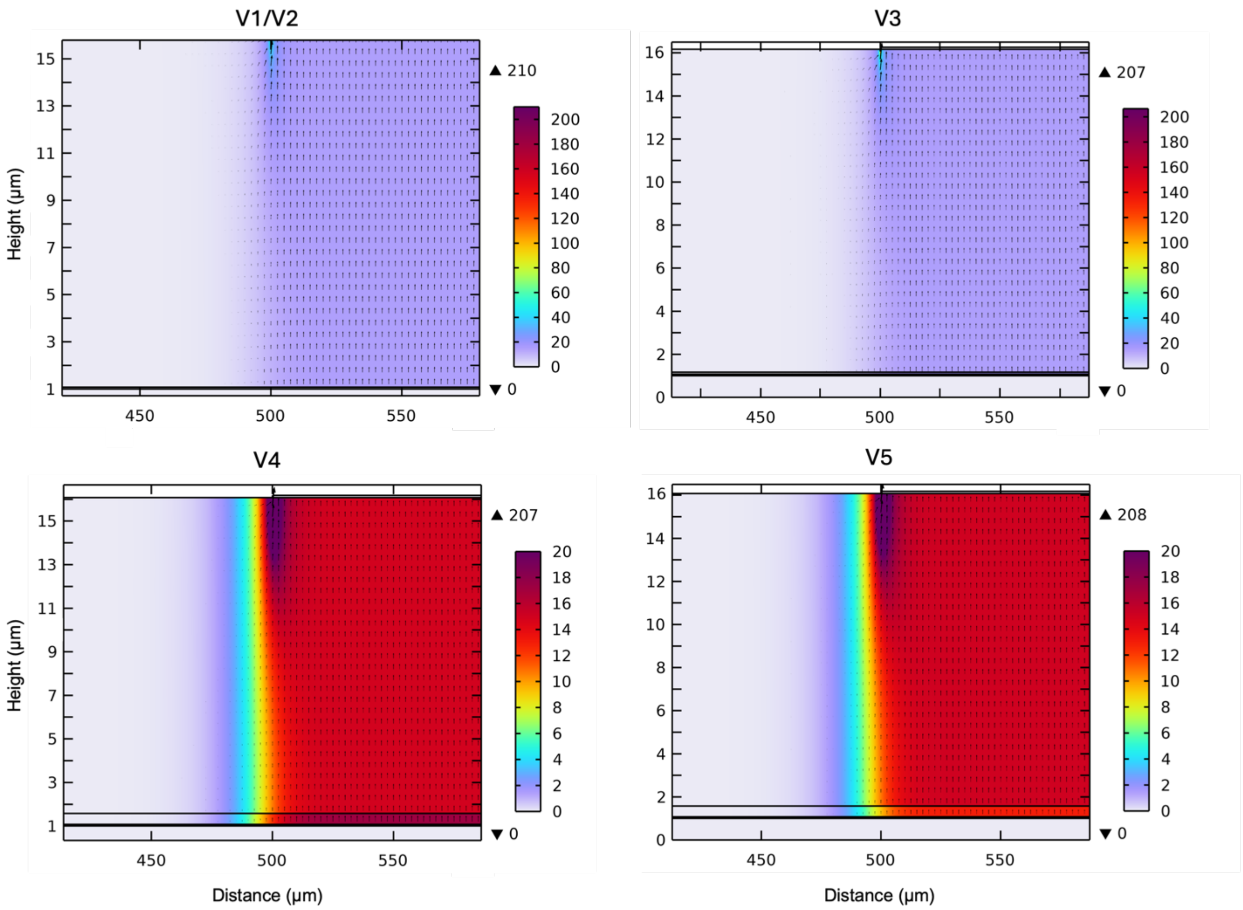

Figure S4. COMSOL models for devices V1/V2, V3, V4, and V5 showing the region between the edge of the top gold electrode and the bottom ITO electrode. The top figures for V1/V2 and V3 are color scaled to show no difference in edge effects around the top electrode, except for a slight deviation in the maximum field spiked in that region. The bottom figures for V4 and V5 are color scaled to show the change in field between the a-Se/Se-Te layers used in the devices.

Vertical devices were simulated with a glass/ITO substrate, photoconductive layers according to the architectures specified in Figure 1 of the main text, and with a 100 nm gold top contact. The glass/ITO substrate was modeled at 1 um/75 nm thickness, respectively, and at 1 mm wide; the photoconductive layer stretched this same width. The top contact covered half this width, beginning half-way through the device. As the circular contacts of our devices are symmetrical through the cross section, only one edge was evaluated. Devices V1 and V2 showed no difference

in electric field behavior or measurement, with a field of 15.0 V/um in the bulk of the material, and so are represented by a single plot. This can be compared against V3, which shows a similar edge effect, seen in all the device models, with variance only in the maximum field spiked at the electrode edge interface. V4 shows the increased field across the thin a-Se layer along the ITO edge, reaching 17.3 V/um, and the slightly reduced field across the Se-Te layer of 14.9 V/um. Figure V5 shows the reduced field across the thin layer of Se-Te of 13.0 V/um, with the thick a-Se layer maintaining a field of 15.1 V/um. For all devices, the electric field drops to 0 V/um within 50 um of the edge region of the top electrode, and becomes negligible within 20 um of this border region. In the bulk of the active area of the device, the fields given above maintain a constant value.

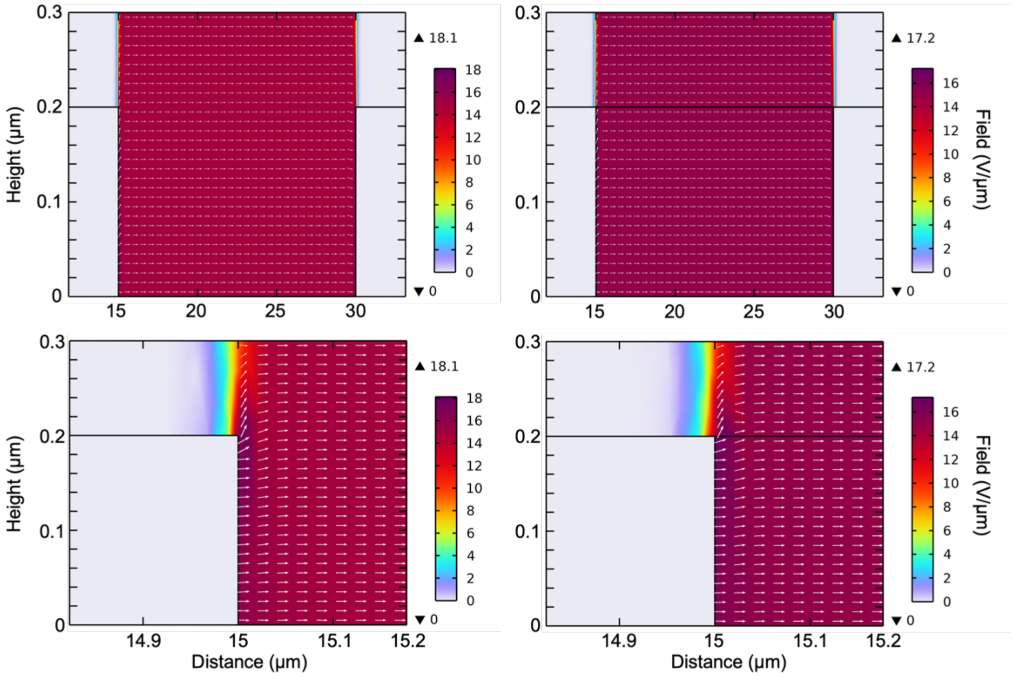

Figure S5. COMSOL models for devices L1/L2 (left) and L3 (right) between two electrodes and around the positive electrode. Each simulation was performed with 225 V applied bias. Top shows the model between two electrodes; bottom shows the area focused around the positive

electrode. Color scales deviate slightly, as the maximum field at the corner of the electrode varied. The average field between electrodes is 15 V/um.

Device L1/L2 consisted of a 300 nm a-Se or Se-Te layer with ε_r=6.3 or 7.7 on top of 15 um wide, 200 nm thick gold electrodes with 15 um spacing; results were indistinguishable, so only one set of plots is shown. Device L3 consisted of 100 nm a-Se on 200 nm Se-Te on the same electrode setup, each with the above dielectric value. All devices were simulated with an applied bias of 225 V. All devices averaged a field of 15 V/um between electrodes, with a slight increase in field up to 18.1 V/um for the single layer devices and 17.2 V/um for the multilayer device around the top corner of the electrodes due to edge effects. The multilayer device show no significant difference in behavior from the solid devices, with no effects from the interface.
